# Delayed Early Primary Visual Pathway Development in Premature Infants: High Density Electrophysiological Evidence

**DOI:** 10.1371/journal.pone.0107992

**Published:** 2014-09-30

**Authors:** Emmanuel Tremblay, Phetsamone Vannasing, Marie-Sylvie Roy, Francine Lefebvre, Damelan Kombate, Maryse Lassonde, Franco Lepore, Michelle McKerral, Anne Gallagher

**Affiliations:** 1 Centre de Recherche, Centre Hospitalier Universitaire Ste-Justine, Montréal, Québec, Canada; 2 Centre de Recherche en Neuropsychologie et Cognition, Département de Psychologie, Université de Montréal, Montréal, Québec, Canada; 3 Département d′Ophtalmologie, Centre Hospitalier Universitaire Ste-Justine, Montréal, Québec, Canada; Medical University of South Carolina, United States of America

## Abstract

In the past decades, multiple studies have been interested in developmental patterns of the visual system in healthy infants. During the first year of life, differential maturational changes have been observed between the Magnocellular (P) and the Parvocellular (P) visual pathways. However, few studies investigated P and M system development in infants born prematurely. The **aim** of the present study was to characterize P and M system maturational differences between healthy preterm and fullterm infants through a critical period of visual maturation: the first year of life. Using a cross-sectional design, high-density electroencephalogram (EEG) was recorded in 31 healthy preterms and 41 fullterm infants of 3, 6, or 12 months (corrected age for premature babies). Three visual stimulations varying in contrast and spatial frequency were presented to stimulate preferentially the M pathway, the P pathway, or both systems simultaneously during EEG recordings. Results from early visual evoked potentials in response to the stimulation that activates simultaneously both systems revealed longer N1 latencies and smaller P1 amplitudes in preterm infants compared to fullterms. Moreover, preterms showed longer N1 and P1 latencies in response to stimuli assessing the M pathway at 3 months. No differences between preterms and fullterms were found when using the preferential P system stimulation. In order to identify the cerebral generator of each visual response, distributed source analyses were computed in 12-month-old infants using LORETA. Source analysis demonstrated an activation of the parietal dorsal region in fullterm infants, in response to the preferential M pathway, which was not seen in the preterms. Overall, these findings suggest that the Magnocellular pathway development is affected in premature infants. Although our VEP results suggest that premature children overcome, at least partially, the visual developmental delay with time, source analyses reveal abnormal brain activation of the Magnocellular pathway at 12 months of age.

## Introduction

In the past decades, several studies have been interested in developmental patterns of the visual system in fullterm and premature infants. In fullterms, a relationship has been demonstrated between specific visual stimulations and preferential parvocellular (P) and magnocellular (M) visual system activation [1] [2]. The P pathway, so-called ventral stream due to anatomical association to the temporal lobe, responds optimally to higher luminance contrasts and is most sensitive to lower temporal and higher spatial frequencies. This pathway is specific to central vision as well as colour and form perception. The M pathway, also known as the dorsal stream because of its structural association to the parietal lobe, responds to lower luminance contrasts and is most sensitive to higher temporal and lower spatial frequencies. It is specific to peripheral vision and processing of rapid motion [3].

During the first year of life, differential maturational changes have been observed between the P and M visual systems. In a previous study, we showed in fullterm infants aged between 0 and 52 weeks that the M pathway matures faster and is functional earlier than the P system [2]. Moreover, the developmental period between 3 and 6 months of age appears to be critical for the maturation of these pathways [1], [2].

The preferential activations of M and P systems can be studied in babies born prematurely with the use of visual evoked potentials (VEPs), more specifically the N1 and P1 components, to investigate the possibility of altered activity in the visual system. VEPs are an objective, non-invasive measure of the electrophysiological response of the occipital cortex following the presentation of a visual stimulus. The literature regarding the developmental pattern in preterm infants, as demonstrated using VEPs and psychophysical measures, is unclear. Some studies suggested an accelerated visual development in age adjusted premature infants with respect to fullterm babies [4], while others found a developmental delay [5].

The current study sought to characterize P and M system maturational differences between healthy preterm and fullterm infants through a critical period of visual maturation, which is the first year of life. We used controlled stimuli, known to preferentially activate either the M or P systems, to evoke N1 and P1 components and applied traditional VEPs peak analyses as well as distributed source models, which have never been used in such a young sample. In order to perform distributed source analyses, dense array electroencephalogram (EEG) was used. Given the methodological limitations, inverse solutions were calculated in 12-month babies only.

## Materials and Methods

### Participants

Thirty-three healthy preterm and 41 fullterm infants were tested using a cross-sectional design at 3, 6, or 12 months of age (see table 1) (corrected ages for preterms). As recommended by the American Academy of Pediatrics [6], preterm age was corrected at 40 weeks' postmenstrual age, according to mothers' reports and ultra- sound examinations before birth [7]. Corrected age represents the age of the child from the expected date of delivery [8] and is calculated by subtracting the number of weeks born before 40 weeks of gestation from the chronological age [6]. The preterm infants were recruited during ophthalmologic assessments included in routine clinical follow-up at the Centre Hospitalier Universitaire Ste-Justine. Reason for prematurity was unknown in most infants. In a few participants, prematurity was due to placental abruption or mother's preclampsy. No participants had major intervention during their stay at the hospital, except for two preterms who underwent a successful Patent Ductus Arteriosus closure. All preterms had their hospital discharge around the expected date of birth or before. The fullterm group infants all had uncomplicated births and were recruited from the obstetric and paediatric units. All participants were in good health at time of testing and had no medical or developmental problems. Complicated pregnancies (i.e. pregnancy diabetes or intra-uterine growth restriction) or children with brain injury or lesion (i.e. periventricular leukomalacia) were excluded from the sample. Any visual problems such as retinopathy of prematurity were ruled out on ophthalmologic assessment. Of these 74 infants, data from two preterm (but none of the fullterms) infants were excluded because of insufficient trials due either to their state of arousal (crying or sleeping babies), movement artefacts, or other non-neurological related activity (e.g., electrical noise, cardiac rhythm artefacts). Data from the remaining 72 participants (31 preterm; 41 fullterm) were included for analysis.

**Table 1:** Demographic and clinical data.

	Preterms			Fullterms		
	3 months	6 months	12 months	3 months	6 months	12 months
N (excluded)	8 (2)	14 (0)	9 (0)	10 (0)	14 (0)	17 (0)
Mean age at testing (mo±SD)	*3.8±0.6*	*6.5±0.6*	*12.7±1.0*	*3.2±0.3*	*6.4±0.6*	*12.0±0.1*
Gestation (w±SD)	*28.9±3.5*	*29.0±3.1*	*29.2±3.2*	*39.1±1.5*	*38.6±1.3*	*38.6±1.2*
Mean weight at birth (g±SD)	*1440.2±595.4*	*1131.9±433.9*	*1272.0±444.6*	*3394.9±524.5*	*3473.4±520.7*	*3419.1±539.1*

N  =  number of participants included in the analyses; mo  =  months; w  =  weeks; SD  =  standard deviation; g  =  grams.

### Ethics statement

The experiment was conducted with the informed written consent of each parent and with formal approval from the CHU Sainte-Justine Ethics and Scientifics committees.

### Stimuli and procedure

**Figure 1:**
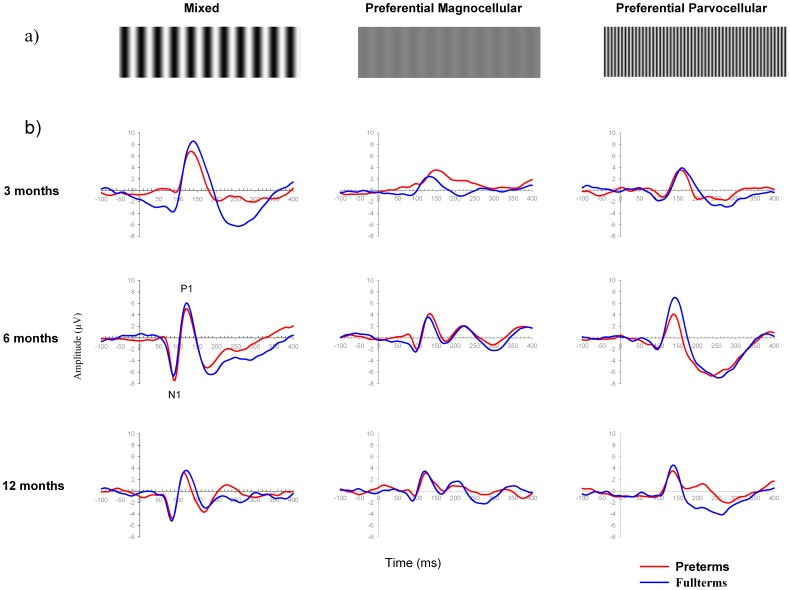
Grand averaged waveforms (Oz electrode) for each stimulus condition and specific age group. (a) Visual stimulations. Left column: mixed or co-activation of P and M pathways (Low95% stimulus); Middle column: preferential M pathway (Low10% stimulus); Right column: the preferential P pathway (High95% stimulus). (b) Grand-average VEP waveforms for preterm (red line) and fullterm (blue line) groups in response to each visual stimulation at 3 (top graph line), 6 (middle graph line), and 12 (bottom graph line) months of age. The same scale was used for each graph in order to appreciate the differential maturational changes in morphology, latency, and amplitude of N1 and P1 components (identify on one graph) according to visual stimulations, ages and groups.

Participants passively viewed a three-phase reversing vertical sine-wave grating of first order covering 20.25×15.25 degrees of visual angle. Stimuli were two low spatial frequencies (0.5 cycles per degree [cpd]) presented at two Michelson [9] contrasts (10% and 95%), and one high spatial frequency (2.5 cpd) at 95% contrast (Figure 1a). A particular stimulus was presented for 500 ms, after which the phase was reversed and lasted for 500 ms (1 Hz), with the original stimulus then appearing, and so on. The current study designated the co-stimulating (i.e. mixed) condition as Low95%, the preferential M system condition as Low10%, and the preferential P system condition as High95%. Luminance remained constant at 30 cd/m2 and shifts between stimuli occurred at a reversal rate of 1 Hz. Each stimulus was presented 150 times. The stimulus sequences were generated by E-Prime Psychology Software (Psychology Software Tools Inc., Pittsburgh, USA), on a DELL computer located in an adjacent room. The recording sessions took place in a dark and soundproof Faraday room. Participants were seated on their parent's lap. In order to keep the participant's gaze onto the stimuli, a small toy was presented in the middle of the monitor by an experienced assistant. In addition, the assistant observed the participant looking at the screen during the whole recording and gave a button press indicator light to the experimenter located into the adjacent computer room whenever the child looked away from the screen. The associated EEG segments were then excluded for further analyses.

### Data acquisition

EEGs were recorded with a high-density Geodesic Sensor Net system with 128 electrodes and were amplified by the Net Amps 200 (Electrical Geodesics Inc., Eugene, OR). Reference was located at the vertex and electrode impedance was kept under 40 kΩ [10]. Ground channel was located on the midline between Cz and Pz (CPz). Using the Net Station program running on a G4 Macintosh computer, EEG signals were acquired at a sampling rate of 250 Hz and analog band pass filtered from 0.1 to 100 Hz.

### Standard EEG data analysis and statistics

EEG signals were analyzed off-line with Brain Vision software, version 2.02 (Brain Vision Products, Munich, Germany). In order to reduce noise (e.g., muscles artefact, drift), the EEG signals were first filtered using a digital band-pass filter (1–30 Hz; *24 dB*/octave) and re-referenced using an average reference [11]. Eye movement artefacts were removed with a semi-automatic ICA method implemented into BrainVision Analyzer [12] using the EGI net default reference channel (Cz). Blink detection was performed using the Value Trigger algorithm to optimize the detection of the prototypical blink patterns. Infomax Extended Biased technique was used for the ICA decomposition (number of ICA steps: 512). For the identification of components related to VEOG and HEOG activity, the Sum of squared Correlation with VEOG/HEOG, which is a correlative score between the component activation and the activity of the selected channels, was used (total value to delete: 60%). For a detailed description of this method see [13]).

VEP time series from −100 ms to 500 ms were edited followed by a semi-automatic artifact rejection based on voltage criteria (±150 µV). EMG electrodes (bilateral temporomandibular joint electrodes) were used as a reference for muscular activity during visual inspection. EEG time series with an amplitude criterion of ±150 µV were withdrawn from the analysis. Because young infants have higher amplitude values compared to older children, this relatively permissive threshold was used to ensure keeping all EEG activity. A visual inspection of data was also applied by an experimented electrophysiologist (P.V.) to remove remaining artifact activity. Good segments were baseline corrected (baseline from −100 to 0 ms) and then averaged. For each subject, EEG time-series were edited in response to the 3 types of visual stimuli: 1) Low95% (co-stimulating condition, mean  = 56 trials (3-month); 66 trials (6-month); 76 trials (12-month) stimuli), 2) Low10% (the preferential M system condition, mean  = 79 trials (3-month); 74 trials (6-month); 81 trials (12-month) stimuli), and 3) High95% (the preferential P system, condition mean  = 51 trials (3-month); 61 trials (6-month); 76 trials (12-month) stimuli).

Examining the topographical distribution of N1 and P1, we identified Oz as the electrode showing the highest VEP amplitude. A semi-automatic peak detection on Oz was thus performed to identify the maximum (P1) and minimum (N1) peaks in a specific time-window (N1 = 60–120 ms; P1 = 100–220). The first negative (N1) and the first positive (P1) components latency and amplitude were identified for each stimulus. Each assessment was reviewed by two experimenters for an inter-rater agreement. Latency and amplitude values were exported to SPSS software for each ERP component. For each visual stimulation condition (Low10%, Low95%, and High95%), analyses of variance (ANOVA) with group (preterms and fullterms) and age (3, 6, and 12 months) were performed on latencies and amplitudes of each component (N1 and P1) separately. Interactions were tested, and Bonferroni post-hoc tests were used. All reported p values used two-tailed tests of significance with set at 0.05. All results are expressed as mean ± standard deviation (SD).

### Source analyses

Partly due to the small head size, white and gray matter segmentation on 3 and 6-month infant brain template was inaccurate. Based on this methodological limitation, inverse solutions were calculated in 12-month babies only.

For each participant, edited single-trial EEGs for each condition were exported to Matlab v7.0.4 (The MathWorks, Inc.) for nonparametric permutation tests and source analyses. In order to determine the time points that were significantly different between the 0 to 500 ms post-stimulus onset time window and the baseline (−100 to 0 ms), a nonparametric permutation test was applied. Nonparametric permutation test was computed on each stimulus condition for both preterm and fullterm infants. Basically, permutation test creates random groups among the two compared conditions (0–500 ms post-stimuli and baseline time windows) while randomly varying their composition. A total of 500 permutations under the null hypothesis of no-difference between conditions were carried out separately on the EEG time series of Oz, O1 and O2 electrodes, where visual responses were expected. The results of the permutation test provided plots of probabilities for accepting the null hypothesis for each sampled time point and for each electrode, defining as significant those time points with probability below the significance level of 0.05 (see [14]; for more details on nonparametric permutation test). Significant time points identified with the permutation test were used for retrieving source analyses on Grand Averaged data.

In order to identify the generators of N1 and P1 components, source analyses using 117 electrodes (i.e., excluding 11 electrodes: 4 VEOG, 2 HEOG, 2 EMG, and 3 additional electrodes located on the nape of the neck (thus not recording cerebral activity) from the 128-electrode set) were computed with low resolution electromagnetic tomography analysis (LORETA)[15] for each significant time points identified using permutation tests. Source analyses were performed using NEURONIC Source Localizer and Tomographic Viewer programs (Neuronic Inc.). Age-matched infant Brain Templates from the Cincinnati Children's Hospital Medical Center [16] were used for the source analyses and preprocessed with the NEURONIC iMagic Pro software (Neuronic Inc.) for extracting the surface of the head and fitting the electrodes to this surface. An isotropic and piecewise homogeneous 3-sphere head model with 18896 (generators) voxels inside the infant's brain with a resolution of 4 mm was used for obtaining the electric lead field [17]. LORETA solutions were first calculated for each visual stimulus condition in every 12-month-old individual subject (preterms and fullterms), and these were averaged across subjects to find the mean LORETA solution for each condition. Specific region of interests (ROI) were defined using Brodmann areas: Occipital area (BA 17), dorso-parietal area (BA 19 and 7), Bilateral temporal cortex (BA 20, 21, 22), and Bilateral frontal areas (BA 10 and 11). Individual source waveforms were extracted from each ROI at N1 and P1 latencies. One-way ANOVAs were performed using SPSS for each visual stimulation condition and ROI in order to identify group differences.

## Results

Although most datasets were normally distributed, a few had an abnormal distribution as shown using Shapiro-Wilk tests. In addition to parametric tests, nonparametric tests were thus performed and provided similar results. In the interests of clarity, only parametric test results are presented here [18].

### VEP latency and amplitude

**Figure 2:**
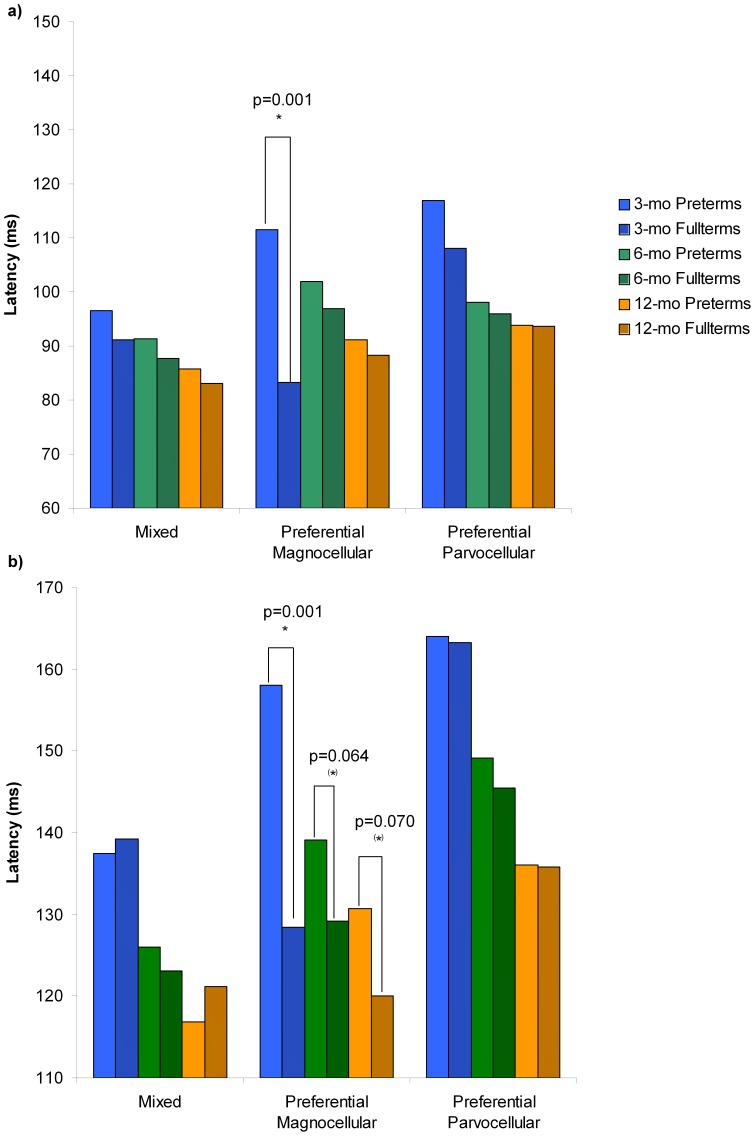
VEPs latencies for each visual stimulation, group and age. On both bar-charts (a and b), VEPs latencies in response to the mixed or co-activations of P and M pathways (Low95% stimulus) are shown on the left part of the histogram; to the preferential M pathway (Low10% stimulus) on the middle part of the histogram; and to the preferential P pathway (High95% stimulus), on the right part of the histogram. For each stimulation, VEP latencies from both groups (preterms and fullterms) and each age (3, 6 and 12 months) are presented separately and identified in the legend. Significant differences (p≤0.05) are identified with *, and tendencies (p>0.05) with (*). a) **N1 component latencies**. In response to the preferential M pathway stimulation, significant differences at 3 months of age are found between preterm and fullterm groups. Moreover, compared to fullterm infants, preterms from all age groups taken together had longer N1 latencies in response to the *Low95% stimulation*, which activate both M and P systems. No differences are found in response to the preferential P pathway stimulations. b) **P1 component latencies**. In response to the preferential M pathway stimulation, significant differences at 3 months of age and statistical tendencies at 6 and 12 months are found between preterm and fullterm groups. No differences are found in response to the mixed and preferential P pathway stimulations.

**Figure 3:**
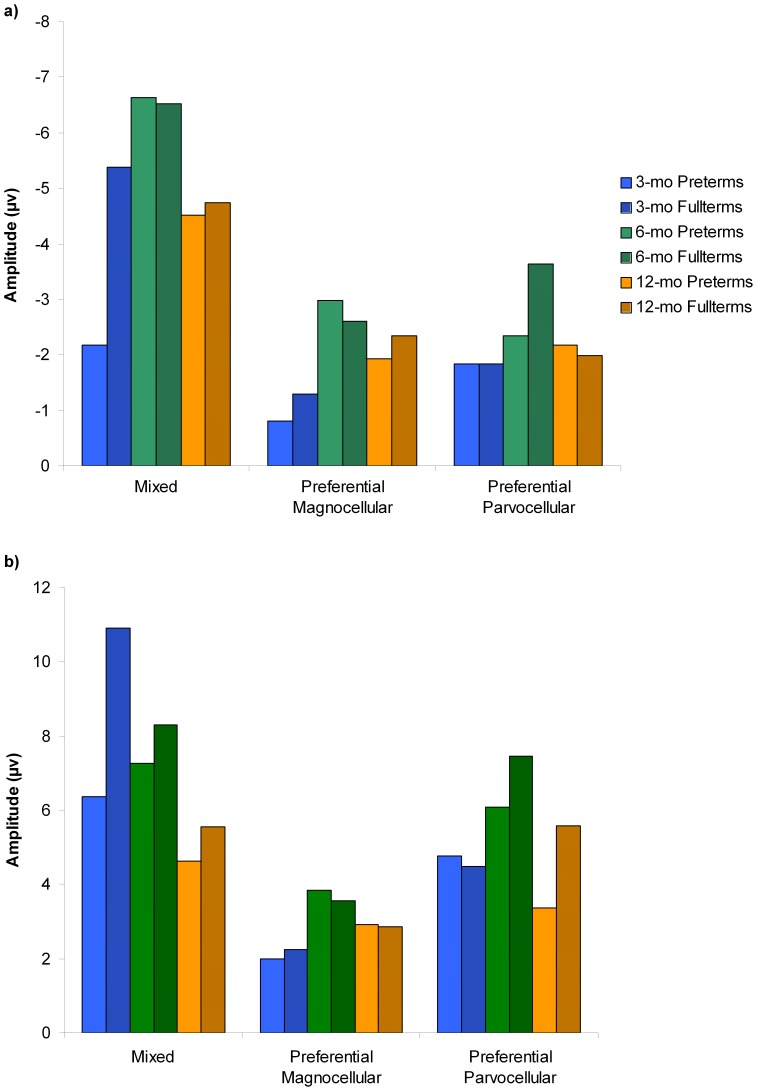
VEPs amplitudes for each visual stimulation, group and age. On both bar-charts (a: N1 component; and b: P1 component), VEPs amplitude in response to the mixed or co-activations of P and M pathways (Low95% stimulus) are shown on the left part of the histogram; to the preferential M pathway (Low10% stimulus) on the middle part of the histogram; and to the preferential P pathway (High95% stimulus), on the right part of the histogram. For each stimulation, VEP amplitudes from both groups (preterms and fullterms) and each age (3, 6 and 12 months) are presented separately and identified in the legend.

Latency and amplitude values of the VEPs (N1 and P1) elicited by each visual stimulus condition were analyzed according to group (preterm and fullterm infants) and age (3, 6, and 12 months) using independent measure ANOVAs. Figure 1b illustrates the grand-average ERPs for preterm and fullterm groups in response to each visual stimulation at 3, 6, and 12 months of age. Figures 2 and 3 show the latency and amplitude values respectively of N1 (a) and P1 (b) for each group and age.

#### Mixed activation of P and M systems (Low95% stimulation)

For latency analyses, significant age and group effects were found on N1, but the age by group interaction was not significant. The age effect (*F*(2,66) = 8.104, *p* = 0.001) reveals that N1 latencies decrease with age (3 months: 93.56±11.73 ms; 6 months: 89.57±5.90 ms; 12 months: 84.00±5.43 ms). The group effect (*F*(1,66) = 4.505, *p* = 0.038) reflects that, taken together, preterms show longer N1 latencies (91.10±9.84 ms) than fullterms (86.63±6.67 ms).

For P1 latencies, only the age effect was significant (*F*(2,66) = 13.651, *p* = 0.001), with P1 latency decreasing with age (3 months: 138.44±14.85 ms, 6 months: 124.57±0.31 ms, 12 months: 119.69±10.97 ms).

For amplitude analyses, a significant age effect was found on N1 (*F*(2,66) = 3.454, *p* = 0.037), amplitude increasing especially between 3 months and 6 months of age (3 months: −3.96±2.92 µV; 6 months: −6.57±4.85 µV; 12 months: −4.65±2.75 µV). No significant group effect or age by group interaction was found.

P1 amplitude was significantly different between groups (*F*(1,66) = 3.929, *p* = 0.052), with greater amplitude in fullterms (7.80±4.66 µV) than in preterms (6.25±4.80 µV), and ages (*F*(2,66) = 3.700, *p* = 0.030), with amplitude decreasing with age (3 months: 8.89±5.25 µV; 6 months: 7.77±5.16 µV; 12 months: 5.23±3.21 µV). No significant group by age interaction was found.

#### The preferential M system (Low10% stimulation)

Latency analyses revealed a significant group by age interaction on N1 (*F*(2,66) = 6.480, p = 0.003) and P1 (*F*(2,66) = 3.113, p = 0.051). Subsequent post-hoc analyses using Bonferroni tests indicated that latencies were significantly delayed in preterm compared to full term infants for N1 (*p* = 0.001; preterm: 111.50±28.16 ms; fullterm: 83.20±11.59 ms) and P1 (*p* = 0.001; preterm: 158.00±27.55 ms; fullterm: 128.40±7.41 ms) at age 3 months. Furthermore, P1 latencies, but not N1 latencies, tended to differ between preterm and full term infants at 6 months (*p = *0.064; preterm: 139.14±16.98 ms; fullterm: 129.14±6.55 ms), and at 12 months (*p* = 0.070; preterm: (130.67±17.09 ms; fullterm: 120.00±6.16 ms).

Amplitude analyses revealed that N1 amplitudes fluctuated with age (*F*(2,66) = 6.261, *p* = 0.003), increasing especially between 3 months and 6 months of age (3 months: −1.08±0.90 µV; 6 months: −2.79±1.94 µV; 12 months: −2.20±1.57 µV). No differences were found between ages for P1 and between groups for N1 and P1.

#### The preferential P system (High95% stimulation)

For latencies analyses, a significant age effect was found on N1 (*F*(2,66) = 5.713, *p* = 0.005) and P1 (*F*(2,66) = 15.314, *p* = 0.001). Both component latencies decrease with age (N1: 3 months: 112.00±23.80 ms; 6 months: 97.00±17.34 ms; 12 months: 93.69±14.85 ms; P1: 3 months: 163.56±20.30 ms; 6 months: 147.29±17.15 ms; 12 months: 135.85±8.80 ms). No group effect or age by group interaction was found.

There were no group or age effects on amplitude values for N1 and P1.

### Source analyses

**Figure 4:**
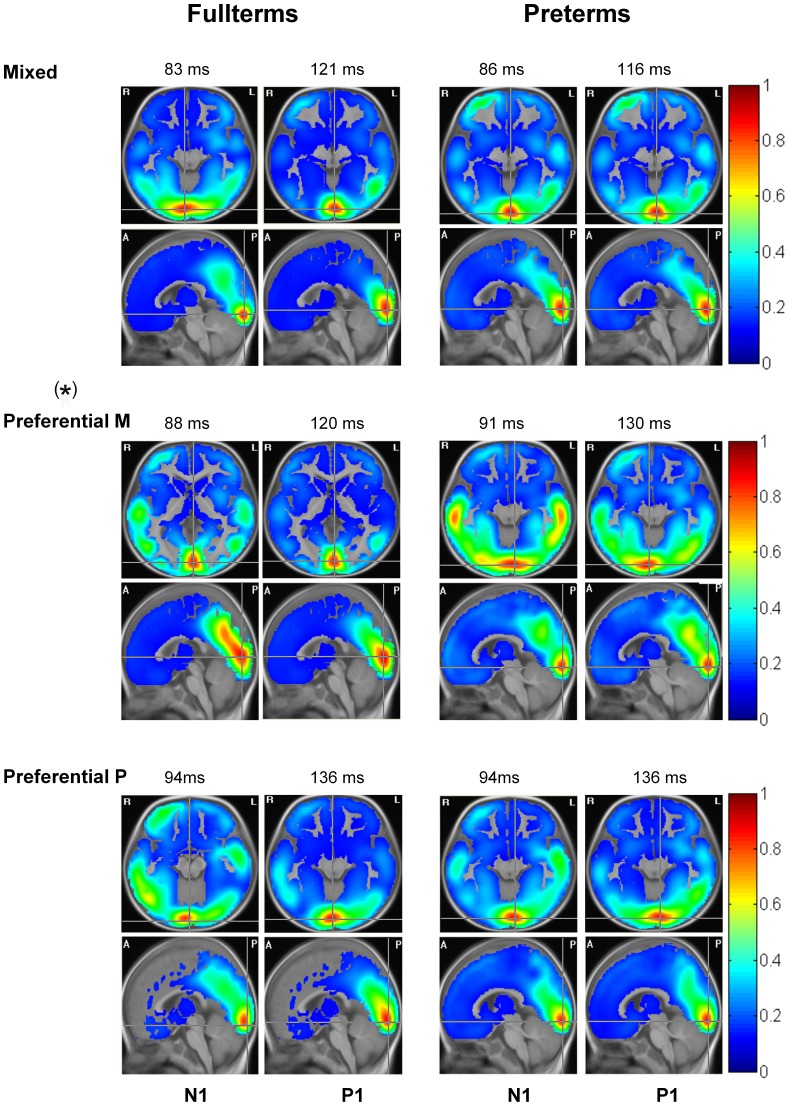
Source distribution. Mean LORETA solutions (represented in transverse and sagittal planes) for the cerebral response induced by three visual stimulations: Mixed (Low95%), which co-activate P and M systems (top line); the preferential M system (Low10%, middle line); and the preferential P system (High95%, bottom line), of 12-month-old fullterms (left column) and preterm (right column). Source distributions at two specific time points, which correspond to time points associated with N1 and P1, are illustrated for each stimulation. (*) Differences on source localisation between fullterms and preterms are seen in response to the preferential M pathway (statistical tendency; p = 0.063). Radiological convention is here used left (L)  =  right (R) and R = L. Anterior (A); posterior (P).

The mean LORETA solutions corresponding to 12-month preterm and fullterm infants cerebral response to each visual stimulus are shown in Figure 4. A permutation test was used to generate latencies for source analysis, by investigating statistical differences between visual stimulus conditions and their own baseline. Source distribution at two specific time points associated with PEVs, which were identified as the two points of maximal difference with the permutation test, are illustrated for each stimulation condition.

Comparison between both groups reveals similar cerebral activation located in the primary visual cortex in 12-month old fullterm and preterm infants at all time points in response to the Low95% (co-activations of P and M systems), Low10% (the preferential M system), and High95% (the preferential P system) stimulations.

For Low95% and High95% conditions, there were no significant differences (p>0.05) between fullterms and preterms for all ROIs at N1 and P1 latencies.

**Figure 5:**
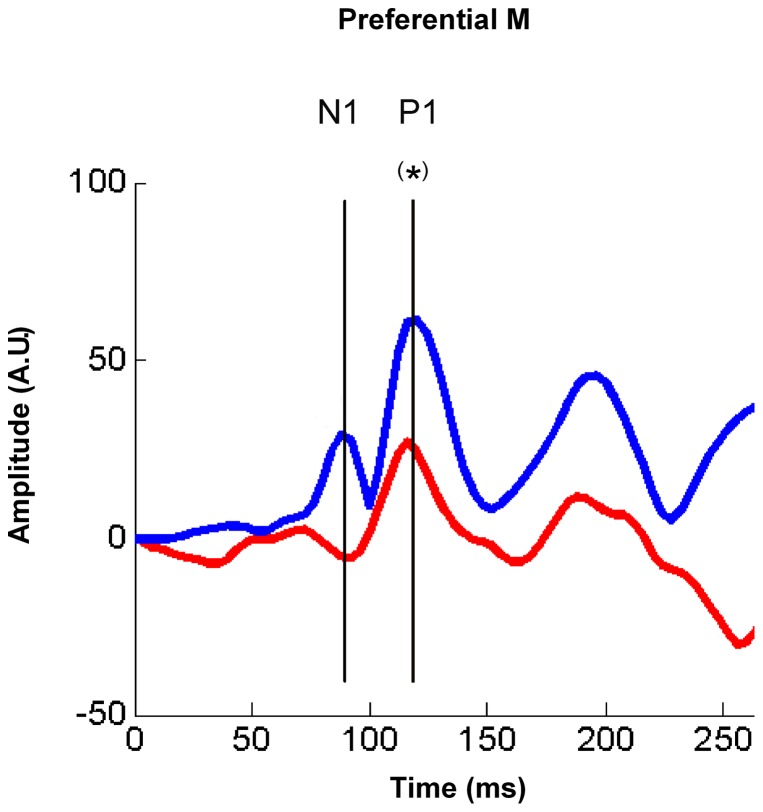
Source waveform intensity for the preferential M system in dorso-parietal areas. Source waveform intensity as a function of time (ms) extracted from the dorso-parietal ROI of both groups (fullterms in blue; preterms in red) for the preferential M system (Low10% visual stimulation). A statistical difference (tendency shown with (*), p = 0.063) is found between groups at 120 ms, corresponding to P1 component. Although a difference between groups is suspected upon visual inspection around 90 ms (N1 component), statistical comparison were not significant, probably because of the low amplitude of this component due to developmental stage at 12 months old. See source distribution of both groups on Figure 4.

In response to Low10% stimulation, a strong activation in the dorso-parietal region was found in fullterm babies, corresponding to the expected mature M pathway response, but not in preterm infants (Figure 4, Preferential M). Source intensity waveforms extracted from the dorso-parietal ROI of both groups are shown on Figure 5. One-way ANOVA revealed a statistical tendency (*F*(1, 27) = 3.788, *p* = 0.063) at 120 ms, which corresponds to the P1 component. No statistical differences were found around the N1 latency. Similarly, no statistical differences were found between groups on source intensity waveforms extracted from bilateral temporal cortex or bilateral frontal areas.

## Discussion

In previous studies, our team as well as others demonstrated that the use of controlled paradigms evoking M or P visual system responses during EEG recordings allowed the electrophysiological identification of the developmental timeline for these pathways [2]. More specifically, the M system is preferentially activated when using low spatial frequency-low contrast stimulus conditions, whereas the P system is predominantly recruited with presentation of high spatial frequency-high visual contrast conditions. In this study, we investigated the impact of premature birth on the development of M and P pathways by studying, in preterm and fullterm infants, the maturation of N1 and P1 VEP components evoked with controlled paradigms and examining brain regions involved when these pathways are activated.

As reported in previous studies [2], the developmental period between 3 and 6 months of age seems to be critical for M and P system development. Indeed, our results show decrease of N1 and P1 latencies with age, especially between 3 and 6 months, confirming the presence of this critical developmental period in fullterm and preterm infants. Moreover, N1 amplitude increases with age, again more importantly between 3 and 6 months. This critically developmental stage probably corresponds to the period of myelinisation of the geniculate-occipital pathway [19].

Our results also reveal that prematurity affects visual system development, more specifically the M pathway. When compared to fullterms, preterm infants show longer N1 and P1 latencies in response to the preferential M system stimulation (*Low10%*). This was found only in 3-month infants for N1 and P1, although a tendency was measured for P1 in 6 and 12-months old participants. Moreover, compared to fullterm infants, preterms had longer N1 latencies and smaller P1 amplitudes in response to the *Low95% stimulation*, which activates both M and P systems. Finally, no difference between preterm and fullterm infants was found when using the preferential P system stimulation (*High95%*). Therefore, our findings suggest that preterm infants have a developmental delay for the M system but not the P system, and that this delay seems to resolve gradually with age. It confirms the developmental vulnerability of the M pathway, compared to the P pathway, in preterm infants reported in previous studies [1], which also showed that prematurity appears to disrupt the development of M visual pathway, in preterm infants aged between 16 and 52 weeks compared to fullterm babies.

A first explanation for these results would be that the M (dorsal) system is more affected by a prematurity than the P system. This possible vulnerability of the M pathway has been reported in a wide range of both genetic and acquired developmental disorders, such as autism [20], dyslexia [21] and Williams syndrome [22]. In the context of premature birth, this vulnerability could be explained by differences in developmental periods for each visual pathway. Indeed, the M pathway development is thought to occur earlier, before birth in the intrauterine environment more precisely during the third trimester of the pregnancy, whereas the P system is thought to develop several weeks later, after birth during neonatal period in fullterms [2], explaining why a premature birth affects more significantly the M visual pathway the P system [1].

Another hypothesis that might explain developmental delay differences between the M and P pathways in preterms is the « visual experience hypothesis » [23]. This hypothesis states that because they are born earlier, preterm infants have more visual experience (coming from the extrauterine world) compared to age-matched fullterm infants. This additional visual experience might accelerate the visual maturation of the P pathway. Bosworth and Dobkins [23], [24] showed that in healthy preterm infants, P visual system maturation is positively influenced by the extra visual experience, but no significant effect was reported on the M system. Thus, the additional visual experience may compensate for the negative effect of prematurity on the P pathway, but not on the M pathway, which might explain why we found a maturational delay for M, but not P, system. In sum, both hypotheses support the present results showing a developmental delay in M, but not in P, visual pathway in preterms, and the explanation might include both hypotheses.

In our group of preterms, M pathway impairments seem to resolve partly with age. This has been previously reported by Bosworth et al. [23]. Using psychophysical assessment, they showed significant developmental delay in the M pathway in 2- to 6- month-old preterm infants, but no more impairment in infants aged between 6 and 11 months, suggesting a gradual resolution of the developmental delay with age. In the present study, we used high-density EEG recordings that allowed distributed sources analyses. Although results from traditional VEPs latency and amplitude peak (at Oz) analyses suggest that the M system maturational delay gradually resolves with age, source analyses show that immature development does not completely disappear and is still in some way present at 12 months in preterm infants. Indeed, the M system recruits the primary visual cortex response in both groups, but only fullterm infants tend to present a stronger activation in the dorso-parietal region, corresponding to the expected mature M pathway response. In addition, an activation is seen in bilateral temporal areas in preterms, compared to fullterm infants. However, no statistical differences were found between groups in this area. Inter-individual variability might explain this non-significant result. This result could also be due to the effect of multiple sources limitations associated with the methodological constraints (spherical head geometry instead of realistic model, use of 12-month-old template instead of individual magnetic resonance imaging) [25]. For the P visual pathway, no difference in brain sources was found. Overall, these results suggest that in 12-month-old preterms, the ventral stream (occipito-temporal or P pathway) is normally developed, whereas the dorsal stream (occipito-parietal or M pathway) is still immature or altered. The use of high-density EEG and distributed source models allowed to investigate changes during development in brain generators of visual systems in our population, showing abnormal M system cerebral activity in response to visual stimulation in 12-month preterm infants.

### Limitations

The present study has several limitations. First, it includes a relatively small sample size. Larger groups of infants would have allowed to investigate factors associated with prematurity, such as birth weight and gestational age, and look at the effect of these factors by dividing infants into several groups (e.g. low birth weight vs. extremely low birth weight; or late, moderate, severe, and extreme prematurity). Another limitation is related to the cross-sectional design of our study. Conducting a longitudinal study can be challenging especially when it comes to participants' compliance. It has not been possible to retrace or recruit participants seen at 3 months of age for multiple testing, explaining the choice of our study design.

## Conclusions

The use of specific VEP paradigms allowed the study of the impact of premature birth on visual development. More specifically, observations of the P1 and N1 components in the Low10% and Low95% conditions show that at 3 months, a delay is present. Since no differences were observed in the High95% condition, the differences observed in the mixed and M condition may reflect an immaturity of the M visual system development. As shown previously [26], these results suggest the absence of benefit of the extra-uterine experience, at least for the M pathway, which may not be the case for the P pathway [4]. It also confirms the maturation vulnerability of the M pathway to prematurity. In spite of the VEP amplitude and latency recovery, the results of the source analyses show that the developmental delay of the M system persists at least until 12 months of age in preterm babies. Further source analysis studies in older children are needed to investigate the M pathway impairment in preterm infants to determine if the M system eventually recruits the parietal region or if it develops abnormally, not recruiting these dorsal regions.

## References

[1] Hammarrenger B, Roy MS, Ellemberg D, Labrosse M, Orquin J, et al (2007) Developmental delay and magnocellular visual pathway function in very-low-birthweight preterm infants. Dev Med Child Neurol 49: 28–33. 17209973 10.1017/s0012162207000084.x

[2] Hammarrenger B, Lepore F, Lippe S, Labrosse M, Guillemot JP, et al (2003) Magnocellular and parvocellular developmental course in infants during the first year of life. Doc Ophthalmol 107: 225–233. 14711154 10.1023/b:doop.0000005331.66114.05

[3] Livingstone M, Hubel D (1988) Segregation of form, color, movement, and depth: anatomy, physiology, and perception. Science 240: 740–749. 3283936 10.1126/science.3283936

[4] Tsuneishi S, Casaer P (2000) Effects of preterm extrauterine visual experience on the development of the human visual system: a flash VEP study. Dev Med Child Neurol 42: 663–668. 11085293 10.1017/s0012162200001225

[5] Braddick O, Atkinson J, Wattam-Bell J (2011) VERP and brain imaging for identifying levels of visual dorsal and ventral stream function in typical and preterm infants. Prog Brain Res 189: 95–111. 21489385 10.1016/B978-0-444-53884-0.00020-8

[6] Engle WA (2004) Age terminology during the perinatal period. Pediatrics 114: 1362–1364. 15520122 10.1542/peds.2004-1915

[7] Wilson-Ching M, Pascoe L, Doyle LW, Anderson PJ (2014) Effects of correcting for prematurity on cognitive test scores in childhood. J Paediatr Child Health 50: 182–188. 24617343 10.1111/jpc.12475

[8] DiPietro JA, Allen MC (1991) Estimation of gestational age: implications for developmental research. Child Dev 62: 1184–1199. 1756662

[9] Michelson A (1927) Studies in Optics. University of Chicago Press.

[10] Tucker DM (1993) Spatial sampling of head electrical fields: the geodesic sensor net. Electroencephalogr Clin Neurophysiol 87: 154–163. 7691542 10.1016/0013-4694(93)90121-b

[11] Nunez PL (1981) A study of origins of the time dependencies of scalp EEG: ii–experimental support of theory. IEEE Trans Biomed Eng 28: 281–288. 7228074 10.1109/TBME.1981.324701

[12] Jung TP, al e (1998) Extended ICA removes artifacts from electroencephalographic recordings. Advances in Neural Information Processing Systems 10: 894–900.

[13] Plank M (2013) Ocular correction ICA. Brain Product Press Release 49: 1–4.

[14] Beauchemin M, Gonzalez-Frankenberger B, Tremblay J, Vannasing P, Martinez-Montes E, et al (2011) Mother and stranger: an electrophysiological study of voice processing in newborns. Cereb Cortex 21: 1705–1711. 21149849 10.1093/cercor/bhq242

[15] Pascual-Marqui RD, Michel CM, Lehmann D (1994) Low resolution electromagnetic tomography: a new method for localizing electrical activity in the brain. Int J Psychophysiol 18: 49–65. 7876038 10.1016/0167-8760(84)90014-x

[16] Altaye M, Holland SK, Wilke M, Gaser C (2008) Infant brain probability templates for MRI segmentation and normalization. Neuroimage 43: 721–730. 18761410 10.1016/j.neuroimage.2008.07.060 PMC2610429

[17] Riera JJ, Fuentes ME (1998) Electric lead field for a piecewise homogeneous volume conductor model of the head. IEEE Trans Biomed Eng 45: 746–753. 9609939 10.1109/10.678609

[18] Kiebel SJ, Tallon-Baudry C, Friston KJ (2005) Parametric analysis of oscillatory activity as measured with EEG/MEG. Hum Brain Mapp 26: 170–177. 15929085 10.1002/hbm.20153 PMC6871741

[19] Yakovlev PI, Lecours AR (1967) The myelogenetic cycles of regional maturation of the brain. In: Minkowski A, editor. Regional development of the brain in early life. Oxford: Blackwell Scientific Publications. pp. 3–70.

[20] Spencer J, O′Brien J, Riggs K, Braddick O, Atkinson J, et al (2000) Motion processing in autism: evidence for a dorsal stream deficiency. Neuroreport 11: 2765–2767. 10976959 10.1097/00001756-200008210-00031

[21] Stein J, Walsh V (1997) To see but not to read; the magnocellular theory of dyslexia. Trends Neurosci 20: 147–152. 9106353 10.1016/s0166-2236(96)01005-3

[22] Atkinson J, Braddick O, Rose FE, Searcy YM, Wattam-Bell J, et al (2006) Dorsal-stream motion processing deficits persist into adulthood in Williams syndrome. Neuropsychologia 44: 828–833. 16168445 10.1016/j.neuropsychologia.2005.08.002

[23] Bosworth RG, Dobkins KR (2013) Effects of prematurity on the development of contrast sensitivity: testing the visual experience hypothesis. Vision Res 82: 31–41. 23485427 10.1016/j.visres.2013.02.009 PMC3684210

[24] Bosworth RG, Dobkins KR (2009) Chromatic and luminance contrast sensitivity in fullterm and preterm infants. J Vis 9: 15 11–16. 10.1167/9.13.15 PMC2932653 20055548

[25] Pizzagalli DA (2007) Electroencephalography and High-Density Electrophysiological Source Localization. In: Cacioppo J.T TLG, Berntson G., editor. Handbook of Phychophhysiology. 3rd edition ed. Cambridge: Cambridge University Press. pp. 56–84.

[26] Grose J, Harding GFA, Wilton AY, Bissenden JG (1999) The maturation of the pattern reversal VEP and flash ERG in pre-term infants. Clinical Vision Sciences 4: 239–249.

